# Update to Trial Forge Guidance 2: addition of the Value of Information criterion

**DOI:** 10.1186/s13063-026-09443-7

**Published:** 2026-01-24

**Authors:** Athanasios Gkekas, Adwoa Parker, Shaun Treweek, Elizabeth Coleman, Catherine Arundel, Frances Shiely

**Affiliations:** 1 York Trials Unit, Department of Health Sciences, University of York, York, UK; 2 Aberdeen Centre for Evaluation, University of Aberdeen, Aberdeen, Scotland UK; 3 School of Public Health, University College Cork, Cork, Ireland; 4 Health Research Board Clinical Research Facility, University College Cork, Cork, Ireland; 5 HRB Trials Methodology Research Network, University College Cork, Cork, Ireland

**Keywords:** Trial Forge Guidance 2, Studies Within A Trial, SWATs, Value of Information Analysis, Recruitment, Retention, Trial processes

## Abstract

Trial Forge Guidance 2 helps trial teams decide if another Study Within A Trial (SWAT) is needed to answer an existing research question. Five criteria are listed to assist decision-making. We are adding a sixth criterion, the Value of Information, to additionally consider the potential time and financial constraints of improving trial process evidence. The Value of Information criterion assesses each trial process based on its value of additional research and value of implementation. We consider evaluations of recruitment and retention strategies as exemplars of how such an approach could be applied to randomised evaluations of trial processes via SWATs. We applied the Value of Information Analysis to all recruitment strategies and categorised them according to their expected benefits in terms of improved recruitment, as well as the level of statistical uncertainty compared to other recruitment strategies. The same approach was followed for retention strategies. To support its use, we have developed an electronic tool that calculates and presents the criterion results for each available evaluation of recruitment and retention strategies. This will enable trial teams to apply the Value of Information criterion, along with the five existing criteria of Trial Forge Guidance 2, when deciding if further SWATs should be prioritised for a particular trial process strategy to provide evidence for the strategy’s use, or not during a trial.

**Supplementary Information:**

The online version contains supplementary material available at 10.1186/s13063-026-09443-7.

## The addition of a Value of Information criterion for Trial Forge Guidance 2

Trial Forge Guidance 2 assists trial teams and researchers in deciding whether further Studies Within A Trial (SWATs) should be undertaken for a trial process strategy [1]. These decisions are currently made using five criteria: Grading of Recommendations, Assessment, Development and Evaluations (GRADE) [1]; a cumulative meta-analysis of the evidence; PICOT (Patient, Intervention, Comparison, Outcome and Time) of existing replications; balance of benefit and disadvantage to participants; and balance of benefit and disadvantage to the host trial [1]. While these criteria cover important aspects surrounding the uncertainty of evidence about a strategy, due to the sparseness of the trial process evidence base, it is unlikely that applying the five criteria to a body of evidence would lead to a decision not to undertake a further evaluation [1].

Thus, in order to additionally consider the potential time and financial constraints of improving trial process evidence, we introduce a sixth criterion, based on a Value of Information Analysis framework, that focuses on the extent of statistical uncertainty presented in meta-analysis results of each trial process evaluation [2]. This framework, which relies on a joint distribution of baseline risk and relative measures of effect (e.g. risk ratio), provides a rigorous approach for evaluating whether the expected benefits of implementing a trial process strategy, given the current level of uncertainty, outweigh its potential negative consequences as a result of the uncertainty (e.g. potentially lower intervention adherence rates relative to a comparator strategy). This approach can quantify the expected value of reducing the uncertainty about the effectiveness of a trial process strategy. Therefore, this new criterion can help trial teams and research commissioners identify which SWAT interventions should be prioritised for additional research, considering the potential time and budget constraints associated with conducting a SWAT in a given setting. If a SWAT is funded and undertaken for a strategy with low uncertainty, this may imply that another SWAT addressing a strategy with greater uncertainty is not conducted, which could incur a high opportunity cost and lead to a misallocation of budget and time resources.

## Methods

In this Trial Forge Guidance 2 update, for illustration purposes, we have applied the Value of Information Analysis framework to strategies exploring two trial processes: participant recruitment and participant retention. We considered randomised SWAT evaluations of 53 recruitment and 51 retention strategies, for which meta-analysis results are available from the ongoing update of the 2018 Cochrane systematic review of strategies to improve trial recruitment and the 2021 Cochrane systematic review of strategies to improve trial retention [3, 4]. In the context of participant recruitment and retention, application of this framework generated the following indicative outputs:

- The value of implementation: the expected number of additional participants recruited (or retained) if the strategy under evaluation is immediately adopted for recruitment (or retention), given its underlying uncertainty, out of a hypothetical population of 1000 trial participants.
- The value of additional research: the expected number of additional participants not recruited (or not retained) due to the uncertainty surrounding the effectiveness of a strategy, out of a hypothetical population of 1000 trial participants.

To estimate these outputs, we extracted meta-analysis data from the Cochrane systematic reviews of strategies to improve recruitment or retention to randomised trials, by expressing relative effect sizes as risk ratios [3, 4]. Taking recruitment, for each evaluation we randomly sampled values for the risk ratio (within the 95% confidence interval for each strategy) and plausible values for baseline recruitment rates (i.e. within the range of 10–50% based on the trial experiences of the authors of the Cochrane review of recruitment strategies [3]), simultaneously for 5000 iterations. For each iteration we multiplied the figure by 1000, which corresponds to the number of hypothetical trial participants, to present these outputs in a standardised manner. This approach generated joint distributions of the risk ratio and baseline recruitment rates, which informs the extent of effect uncertainty in each recruitment strategy and thus provides estimates for the two outputs of interest; in order to model uncertainty, we assumed independence between the two parameters (risk ratio and baseline recruitment rate). The same approach was taken for retention strategies except we used plausible baseline retention rates of 50 to 80%, based on the trial experiences of the authors of the Cochrane review of retention strategies [4] and the range of retention rates reported across UK-based randomised trials [5]. Similarly, risk ratios and baseline risks for an alternative trial process strategy would also need to be identified. Methodological details about the application of the Value of Information framework in the context of SWAT interventions can be found in Supplementary Material 1.

The Value of Information criterion categorises each trial process strategy based on its value of additional research and value of implementation, resulting in four possible categories. These are presented in Table 1 under the trial processes of participant recruitment and retention. Each strategy is assessed on two factors: (1) whether it can yield positive or negative expected benefits in terms of the trial process under evaluation, e.g. recruitment or retention, and (2) whether its underlying uncertainty is more or less pronounced compared to other strategies targeting the same trial process.

**Table 1:** Value of Information criterion

Category	Statistical description	Description for TFG2	Value of Information criterion met
A	• > Median value of additional research • Value of implementation ≤ 0	Current evidence suggests a low likelihood of the strategy offering incremental benefits for the trial process under evaluation. Further research is suggested for this strategy due to significant uncertainty regarding its effectiveness, in relation to comparisons of other strategies targeting the trial process under evaluation.	Yes
B	• < Median value of additional research • Value of implementation ≤ 0	Current evidence suggests a low likelihood of the strategy offering incremental benefits for the trial process under evaluation. Underlying uncertainty in the evidence of this strategy is less pronounced in relation to comparisons of other strategies targeting the trial process under evaluation.	No
C	• > Median value of additional research • Value of implementation > 0	Current evidence suggests a high likelihood of the strategy offering incremental benefits for the trial process under evaluation. Further research is suggested for this strategy due to significant uncertainty regarding its effectiveness, in relation to comparisons of other strategies targeting the trial process under evaluation.	Yes
D	• < Median value of additional research • Value of implementation > 0	Current evidence suggests a high likelihood of the strategy offering incremental benefits for the trial process under evaluation. Underlying uncertainty in the evidence of this strategy is less pronounced in relation to comparisons of other strategies targeting the trial process under evaluation.	No

The Value of Information criterion is met if a strategy falls into category A or C. If met, it means it may be relatively valuable for research commissioners to fund further SWAT-related research in evaluations of this strategy, as its underlying effect uncertainty is relatively high compared to other strategies targeting the same trial process. For trial teams, it means that undertaking a SWAT on such a strategy could improve the evidence base and effectively help reduce the uncertainty surrounding the trial process under evaluation. Instead, if the criterion is not met, it may not be a priority for research commissioners to fund further SWATs of such a strategy because the underlying uncertainty is relatively low compared to other strategies. For trial teams, it means it may be more valuable to improve the evidence base by undertaking SWATs of alternative strategies that present higher effect uncertainty.

Under the trial process of participant recruitment, the benchmark we established to determine whether additional SWAT research is recommended was based on the current median value of additional research across all recruitment strategies. This value currently stands at 4.71 per 1000 trial participants, meaning that further research on a recruitment strategy would be encouraged if it could yield additional recruitment benefits of at least 4.71 per 1000 trial participants, a figure that is surpassed by half of the existing recruitment strategies. The corresponding figure for retention strategies is 3.72. Similarly, for other types of trial processes, a median value of additional research across all strategies for a given trial process should be established as a benchmark.

We have developed an electronic tool which includes the Value of Information criterion results for all evaluations of recruitment (and retention) strategies presented in the two Cochrane systematic reviews. This tool has been reviewed by funders of trials, trialists, and trial methodologists, who responded to a brief questionnaire (Supplementary Material 2); their feedback was used to refine the tool by adding instructions to users on how to use the tool and improving the descriptions of the outputs. If trial teams need to decide whether to conduct another SWAT for recruitment (or retention), they can insert confidence intervals (in terms of risk ratio) for a recruitment (or retention) strategy developed in the future, which will produce estimates for its value of additional research, its value of implementation, and consequently for its Value of Information criterion. The outputs from the tool should be considered with the other five criteria from Trial Forge Guidance 2. To assist decision-makers in the absence of a definitive threshold for the value of additional research, the electronic tool also ranks the uncertainty of recruitment (or retention) strategies, in descending order, based on their value of additional research (per 1000 trial participants). This tool has been developed with Microsoft Excel and is available on the Trial Forge website [6].

## Application of the Value of Information criterion

As in the initial Trial Forge Guidance 2 paper [1], we use the same working examples to illustrate how to apply the Value of Information criterion: telephone reminders versus no telephone reminders (a recruitment strategy) and monetary incentives versus no monetary incentives (a retention strategy). As both examples present statistical significance, we also add a strategy that is non-statistically significant for illustrative purposes, i.e. optimised information versus standard information (a retention strategy). The findings are summarised in Table 2 and presented in a similar format to that used in the electronic tool. The forest plots related to the three strategies, expressed in terms of risk ratios, can be found in Supplementary Material 3.

**Table 2:** Application of the Value of Information criterion

Output	Value of the output	Description of output
Example 1: telephone reminders versus no telephone reminders (a recruitment strategy)		
Risk ratio (RR) 95% confidence interval	1.25 to 3.02	Reported confidence intervals from meta-analysis data extracted from the Cochrane systematic review of strategies to improve recruitment to randomised trials [3]
Value of additional research (per 1000 trial participants)	0	The additional potential participants recruited (per 1000) if the associated uncertainty of the recruitment strategy under evaluation was reduced
Value of implementation (per 1000 trial participants)	341.85	The additional potential participants recruited (per 1000), if the recruitment strategy under evaluation was adopted in all relevant randomised trials
Value of information criterion met (Trial Forge Guidance 2)	No	Is the value of information criterion met for Trial Forge Guidance 2? If yes, it means that further research is recommended
What should I do with this information?	Current evidence suggests a high likelihood of the strategy offering incremental benefits with regard to participant recruitment. The underlying uncertainty in the evidence of this strategy is less pronounced in relation to comparisons of other recruitment strategies	The recommendations of the Value of Information criterion should be interpreted alongside the other Trial Forge Guidance 2 criteria
Example 2: monetary incentives versus no monetary incentives (a retention strategy)		
Risk ratio (RR) 95% confidence interval	1.06 to 1.36	Reported confidence intervals from meta-analysis data extracted from the Cochrane systematic review of strategies to improve retention in randomised trials [4]
Value of additional research (per 1000 trial participants)	0	The additional participants retained (per 1000) if the associated uncertainty of the retention strategy under evaluation was reduced
Value of implementation (per 1000 trial participants)	136.89	The additional participants retained (per 1000), if the retention strategy under evaluation was adopted in all relevant randomised trials
Value of information criterion met (Trial Forge Guidance 2)	No	Is the value of information criterion met for Trial Forge Guidance 2? If yes, it means that further research is recommended
What should I do with this information?	Current evidence suggests a high likelihood of the strategy offering incremental benefits with regard to participant retention. The underlying uncertainty in the evidence of this strategy is less pronounced in relation to comparisons of other retention strategies	The recommendations of the Value of Information criterion should be interpreted alongside the other Trial Forge Guidance 2 criteria
Example 3: optimised information versus standard information (a retention strategy)		
Risk ratio (RR) 95% confidence interval	0.85 to 1.09	Reported confidence intervals from meta-analysis data extracted from the Cochrane systematic review of strategies to improve retention in randomised trials [4]
Value of additional research (per 1000 trial participants)	10.76	The additional participants retained (per 1000) if the associated uncertainty of the retention strategy under evaluation was reduced
Value of implementation (per 1000 trial participants)	−19.75	The additional participants retained (per 1000), if the retention strategy under evaluation was adopted in all relevant randomised trials
Value of information criterion met (Trial Forge Guidance 2)	Yes	Is the value of information criterion met for Trial Forge Guidance 2? If yes, it means that further research is recommended
What should I do with this information?	Current evidence suggests a low likelihood of the strategy offering incremental benefits with regard to participant retention. Further research is suggested for this strategy due to significant uncertainty regarding its effectiveness, in relation to comparisons of other retention strategies	The recommendations of the Value of Information criterion should be interpreted alongside the other Trial Forge Guidance 2 criteria

### Example 1: telephone reminders versus no telephone reminders (a recruitment strategy)

The evidence for this strategy arises from two SWATs [7, 8], with an estimated risk ratio of 1.94 (95% CI: 1.25 to 3.02). Following a random sampling of 5000 random combinations of risk ratio (i.e. varying between 1.25 and 3.02) and baseline recruitment rates (varying between 10 and 50%), a joint distribution of risk ratio values and baseline recruitment rates is produced that reflects the effect uncertainty of telephone reminders as a recruitment strategy. Based on this distribution, the value of implementation is 341.85 per 1000 trial participants, which means that 341.85 additional participants, out of 1000 potential trial participants, would be recruited if they received telephone reminders, compared to not receiving them. In addition, as the probability of telephone reminders being an effective recruitment strategy is 100%, which reflects the lower confidence bound of the risk ratio exceeding 1.00, the value of additional research is zero per 1000 trial participants. This means that additional research is not expected to yield additional recruitment benefits by reducing the uncertainty surrounding this evaluation. Given that the median value of additional research for all recruitment strategies is greater than the value of additional research for this strategy (i.e. the median of 4.71 is greater than the zero value of additional research for telephone reminders), and that telephone reminders produce a positive value of implementation, this strategy falls into category D. Thus, the Value of Information criterion is not met, i.e. the value of SWAT-related research would be higher in evaluations of other recruitment strategies whose effect uncertainty is more pronounced compared to telephone reminders versus no telephone reminders.

### Example 2: monetary incentives versus no monetary incentives (a retention strategy)

The evidence for this strategy arises from three randomised SWATs [9–11], with an estimated risk ratio of 1.20 (95% CI: 1.06 to 1.36). Following a random sampling of 5000 random combinations of risk ratio (i.e. varying between 1.06 and 1.36) and baseline retention rates (varying between 50 and 80%), a joint distribution of risk ratio values and baseline retention rates is produced that reflects the effect uncertainty of monetary incentives as a retention strategy. Based on this distribution, the value of implementation is 136.89 per 1000 trial participants, which means that 136.89 additional participants, out of 1000 trial participants, would be retained if they received monetary incentives, compared to not receiving them. In addition, as the probability of monetary incentives being an effective retention strategy is 100%, which reflects the lower confidence bound of the risk ratio exceeding 1.00, the value of additional research is zero per 1000 trial participants, which means that additional research is not expected to yield additional retention benefits by reducing the uncertainty surrounding this evaluation. Given that the median value of additional research for all retention strategies is greater than the value of additional research for this strategy (i.e. the median of 3.72 is greater than the zero value of additional research for monetary incentives), and that monetary incentives produce a positive value of implementation, this strategy falls into category D. Thus, the Value of Information criterion is not met, i.e. the value of SWAT-related research would be higher in evaluations of other retention strategies whose effect uncertainty is more pronounced compared to monetary incentives versus no monetary incentives.

### Example 3: optimised information versus standard information (a retention strategy)

The evidence for this strategy arises from two randomised SWATs, with an estimated risk ratio of 0.96 (95% CI: 0.85 to 1.09) [12, 13]. Following a random sampling of 5000 random combinations of risk ratio (i.e. varying between 0.85 and 1.09) and baseline retention rates (varying between 50 and 80%), the value of implementation is −19.75 per 1000 trial participants, which means that 19.75 fewer participants, out of 1000 trial participants, would be retained if they received optimised information (e.g. trial materials, such as participant information sheets updated following user-testing), compared to standard information (e.g. trial materials, such as standard participant information sheets designed by trial teams). In addition, as the probability of optimised information not being an effective retention strategy is 67%, as 33% of the iterations of the joint distribution produce positive figures, the value of additional research is 10.76 per 1000 trial participants, which means that additional research is expected to yield additional retention benefits (10.76 per 1000 trial participants) by reducing the uncertainty surrounding the effectiveness of standard information. Given that the median value of additional research for all retention strategies is lower than the value of additional research for this strategy (i.e. the median of 3.72 is less than the value of additional research of 10.76), and that optimised information produces a negative value of implementation, this strategy falls into category A. Thus, the Value of Information criterion is met, i.e. further SWAT-related research on the evaluation of optimised information versus standard information would be valuable, due to relatively significant effect uncertainty.

## Discussion

By adding the Value of Information criterion to Trial Forge Guidance 2, we aim to quantify the extent of statistical uncertainty associated with evaluations of trial processes. In the context of recruitment (and retention) strategies, this approach may identify strategies whose uncertainty may have a more significant impact on participant recruitment (or retention) compared to others targeting the same trial process. The Value of Information criterion can also be applied to randomised evaluations of strategies for other trial processes, such as data collection, data management, protocol design, and training trial staff, as long as the outcome measures in such evaluations are quantitative and binary (e.g. consent error rates, intervention adherence rates).

We consider this approach an important addition to the existing Trial Forge Guidance 2 criteria, as it introduces a comparative element that enhances our understanding of which trial process strategies could potentially benefit from further SWAT research. This can be relevant for research teams interested in undertaking SWATs, and research commissioners who are often subject to resource constraints, allowing them to identify SWATs of trial processes that could yield the highest return on investment. As part of this update, we have also introduced an accessible electronic tool for users to apply the Value of Information criterion to evaluations of all existing recruitment and retention strategies, as well as to those of recruitment and retention strategies developed in the future. Our aim is to regularly update this tool as other randomised SWAT evaluations are conducted in recruitment and retention, as well as integrate evaluations of other types of trial processes into this tool.

We encourage the equal consideration of the Value of Information criterion alongside the other five criteria from Trial Forge Guidance 2. For instance, with respect to the existing evaluations of using telephone reminders as a recruitment strategy (versus no telephone reminders), the GRADE criterion highlights the small sample size involved for the cumulative evidence (*n* = 1450), and the moderate GRADE certainty of evidence for randomised trials whose baseline recruitment rate exceeds 10%, whereas the cumulative evidence criterion highlights the low number of included SWATs (two). The Value of Information criterion adds another perspective by concluding that the underlying uncertainty of this strategy is less pronounced compared to that found in evaluations of other recruitment strategies. Thus, whereas additional SWAT research could improve the certainty of the evidence surrounding telephone reminders, it may be more appropriate for trial teams to consider SWAT replications of recruitment strategies with relatively higher effect uncertainty, considering the sparseness of the trial process evidence base. The Value of Information criterion can present the recruitment (and retention) strategies which currently have the highest degree of effect uncertainty.

Nevertheless, we should also highlight that a trial process strategy is estimated to have a value of zero for additional research when the lower bound of the risk ratio confidence interval is equal to or greater than one, regardless of the sample size and the number of SWATs included in a meta-analysis. In other words, even if the value of additional research is zero, conducting further research may still be worthwhile. While conducting additional SWATs might not always be a priority, it could provide a more accurate representation of the actual underlying uncertainty—potentially leading to narrower or wider intervals depending on the prior information derived from previous SWATs—thus enhancing confidence in the robustness of the results, in line with the cumulated evidence criterion. In addition, the benchmark related to the median value of additional research across all strategies within a given trial process is not intended as a definitive threshold. Instead, it was adopted to identify trial process strategies for which additional research could potentially yield the greatest benefits by reducing effect uncertainty, compared to other strategies, while ensuring international comparability. Using median values for additional research avoids the complexities of estimating country-specific SWAT-related research costs and the need to monetise uncertainty. To translate these standardised figures into national-level thresholds, decision-makers from different countries are encouraged to either establish monetary thresholds in their own settings, such as a desired incremental cost per additional participant involved in a trial process (e.g. recruited), or compare SWAT research proposals based on their expected value in improving trial processes. If uncertainty was expressed monetarily, trial teams would need to estimate an annual number of trial participants expected to benefit from an additional SWAT for a given trial process strategy. In this update, we used a hypothetical trial population of 1000 for analytical purposes, to ensure the values of implementation and the values of additional research of different trial process strategies were comparable on a common scale, while assuming that each recruitment or retention strategy could be implemented in all future randomised trials. In addition, trial teams would need to consider the context under which a trial process strategy would be evaluated (e.g. digital versus non-digital participant retention) and the potentially variable research costs these may introduce when drafting a SWAT grant proposal. Finally, trial teams would need to account for efficiency gains in terms of a trial process over a longer time horizon before estimating the value of their research proposal (e.g. £20 per additional participant adhering to trial intervention), as a given trial process strategy may continue to produce benefits over time. Finally, stakeholders need to assess the extent to which each trial process strategy could be implemented across randomised trials; in this update, we assumed that all recruitment and retention strategies could be feasibly implemented across all randomised trials, although in practice the extent of feasibility may vary. Nonetheless, the present Trial Forge Guidance 2 update provides a starting point for identifying strategies likely to be worthwhile for research prioritisation, given the current level of statistical uncertainty, in the absence of specific monetary thresholds.

In conclusion, by incorporating the Value of Information Criterion and considering it equally alongside the other five existing Trial Forge Guidance 2 criteria, trial teams and funders can gain a comprehensive understanding of all factors influencing the need for further research into a trial process strategy, as well as the strengths and limitations of the existing evidence.

## Supplementary Information

Supplementary Material 1.

Supplementary Material 2.

Supplementary Material 3.

## Data Availability

The datasets generated and/or analysed during the current study are available in the Excel spreadsheet published on the Trial Forge website (https://www.trialforge.org/resource/value-of-information-tool/).

## Abbreviations

GRADE: Grading of Recommendations, Assessment, Development and Evaluations
PICOT: Patient, Intervention, Comparison, Outcome and Time
RR: Risk ratio
SWAT: Study Within A Trial
