# Supplementary material for: Update to Trial Forge Guidance 2: addition of the Value of Information criterion
Source: Trials. 2026 Jan 24;27:141. doi: 10.1186/s13063-026-09443-7 (PMC12910824; doi:10.1186/s13063-026-09443-7)
Supplement: Supplementary file 1 — Supplementary Material 1. [file 13063_2026_9443_MOESM1_ESM.docx]

**Supplementary Material 1: Estimating the value of implementation and the value of additional research**

For a given recruitment strategy

- Report the 95% confidence interval lower and upper bounds, based on meta-analysis data extracted from the updated Cochrane systematic review of strategies to improve recruitment to randomised trials. The measure of effect is risk ratio (RR). (RR_recruitment,k_)
- Use an appropriate baseline recruitment rate (it is generated in the electronic tool automatically). We regard this to be a random figure between 10% and 50% (*BaselineRecruitment_k_*)
- The trial population is 1000, corresponding to a hypothetical population of 1000 trial participants. This figure is used for standardisation purposes.
- For the recruitment strategy under evaluation, apply random sampling of K=1,2,..,k,.., 5000 iterations with respect to the distribution of:
  *JointRecruitment_k_= BaselineRecruitment_k_* * (RR_recruitment,k_) * 1000

, in order to generate 5000 values of JointRecruitment_k_ , where k=1,..., 5000 is the labelled number of iteration. Note that RR_recruitment,k_ can take any value between the lower and the upper CI bound in each k iteration. For each k iteration, BaselineRecruitment_k_ also varies since the randomly chosen baseline recruitment rate can take a value of between 10% and 50%. 1000 represents the fixed value for the trial population. We assume that *BaselineRecruitment_k_* and RR_recruitment,k_ are independent.

- For the comparator, apply

*ComparatorRecruitment_k_= BaselineRecruitment_k_* * 1000,

For each k iteration, BaselineRecruitment_k_ varies as the chosen baseline recruitment rate varies between 10% and 50%. 1000 represents the fixed value for the trial population.

- For each k iteration, obtain Effectiveness_k_, which expresses the effectiveness of the recruitment strategy under evaluation.

*Effectiveness_k_ = JointRecruitment_k_* - *ComparatorRecruitment_k_*

, where

- if Effectiveness_k_>0, the strategy is regarded to be effective under iteration k
- If Effectiveness_k_=0, the strategy is regarded to be as effective as the comparator under iteration k.
- if Effectiveness_k_<0, the strategy is regarded to be ineffective under iteration k

- Obtain the frequentist probability of the recruitment strategy being effective. This implies to identify the cases where under the generated distribution Effectiveness_k_>0.

P (Strategy Effective) = $\frac{\sum_{k=1}^{5000} Xk}{k}$

, where for each iteration k, X_k_=1 when Effectiveness_k_>0

X_k_=0 when Effectiveness_k_$\leq$0

- Correspondingly, the probability of the strategy being ineffective is expressed as:

P (Strategy Not Effective) =1- $\frac{\sum_{k=1}^{5000} Xk}{k}$

- **The value of implementation** of a given recruitment strategy is expressed as the number of additional potential participants expected to be recruited to randomised trials because of the recruitment strategy, in relation to the comparator strategy, expressed as the weighted average of Effectiveness_k_ across the 5000 iterations.

ValueOfImplementation_recruitment_= $\frac{\sum_{k=1}^{5000} (Effectivenessk)}{k}$ *δ

, where δ∈(0,1) is the proportion of trials for which such a recruitment strategy could be applied. In our analysis, we assume that for all recruitment strategies, δ=1.

- **The value of additional research** on a given recruitment strategy can be estimated through the weighted average of Effectiveness_k ,_ conditional upon the iterations for which Effectiveness_k_≤0 multiplied by P (Strategy Not Effective). In other words, the value of additional research corresponds to the expected number of potential participants not recruited as a result of the effect uncertainty around the given recruitment strategy, multiplied by the probability of the strategy not being effective:

VOI_recruitment_= $|\frac{\sum_{k=1}^{k} \left( Effectivenessk \right|Effectivenessk<0)}{k}$| * P (Strategy Not Effective) *δ

if ValueOfImplementation_recruitment_ >0

, where δ∈(0,1) and we assume that for all recruitment strategies, δ=1.

However, if ValueOfImplementation_recruitment_ <0, then the value of additional research is estimated as:

VOI_recruitment_= $|\frac{\sum_{k=1}^{k} \left( Effectivenessk \right|Effectivenessk>0)}{k}$| * P (Strategy Effective) *δ

if ValueOfImplementation_recruitment_ $\leq$0

, where δ∈(0,1) and we assume that for all recruitment strategies, δ=1.

For a given retention strategy

- Report the 95% confidence interval lower and upper bounds based on meta-analysis data extracted from the Cochrane systematic review of strategies to improve retention in randomised trials. The measure of effect is risk ratio (RR). (RR_retention,k_)
- Use an appropriate baseline retention rate (it is generated in the electronic tool automatically). We regard this to be a random figure between 50% and 80% (*BaselineRetention_k_*)
- The trial population is 1000, corresponding to a hypothetical population of 1000 trial participants. This figure is used for standardisation purposes.
- For the retention strategy under evaluation, apply random sampling of K=1,2,..,k,.., 5000 iterations with respect to the distribution of:
  *JointRetention_k_= BaselineRetention_k_* * (RR_retention,k_) * 1000

, in order to generate 5000 values of JointRetention_k_ , where k=1,..., 5000 is the labelled number of iteration. Note that RR_retention,k_ can take any value between the lower and the upper CI bound in each k iteration. For each k iteration, BaselineRetention_k_ also varies since the chosen baseline retention rate varies between 50% and 80%. 1000 represents the fixed value for the trial population. We assume that *BaselineRetention_k_* and RR_retention,k_ are independent.

- For the comparator, apply

*ComparatorRetention_k_= BaselineRetention_k_* * 1000,

For each k iteration, BaselineRetention_k_ varies as the chosen baseline retention rate varies between 50% and 80%. 1000 represents the fixed value for the trial population.

- For each k iteration, obtain Effectiveness_k_, which expresses the effectiveness of the retention strategy under evaluation.

*Effectiveness_k_ = JointRetention_k_* - *ComparatorRetention_k_*

, where

- if Effectiveness_k_>0, the strategy is regarded to be effective under iteration k
- If Effectiveness_k_=0, the strategy is regarded to be as effective as the comparator under iteration k.
- if Effectiveness_k_<0, the strategy is regarded to be ineffective under iteration k

- Obtain the frequentist probability of the retention strategy being effective. This implies to identify the cases where under the generated distribution Effectiveness_k_>0.

P (Strategy Effective) = $\frac{\sum_{k=1}^{5000} Xk}{k}$

, where for each iteration k, X_k_=1 when Effectiveness_k_>0

X_k_=0 when Effectiveness_k_$\leq$0

- Correspondingly, the probability of the strategy being ineffective is expressed as:

P (Strategy Not Effective) =1- $\frac{\sum_{k=1}^{5000} Xk}{k}$

- **The value of implementation** of a given retention strategy is expressed as the number of additional participants expected to be retained in randomised trials as a result of the retention strategy, in relation to the comparator strategy, expressed as the weighted average of Effectiveness_k_ across the 5000 iterations.

ValueOfImplementation_retention_= $\frac{\sum_{k=1}^{5000} (Effectivenessk)}{k}$ *δ

, where δ∈(0,1) is the proportion of trials for which such a retention strategy could be used. In our analysis, we assume that for all retention strategies, δ=1.

- **The value of additional research** on a given retention strategy can be estimated through the weighted average of Effectiveness_k,_ conditional upon the iterations for which Effectiveness_k_≤0 multiplied by P (Strategy Not Effective). In other words, the value of additional research corresponds to the expected number of participants not retained as a result of the effect uncertainty around the given retention strategy, multiplied by the probability of the strategy not being effective:

VOI_retention_= $|\frac{\sum_{k=1}^{k} \left( Effectivenessk \right|Effectivenessk<0)}{k}$| * P (Strategy Not Effective) *δ

if ValueOfImplementation_retention_ >0

, where δ∈(0,1) and we assume that for all retention strategies, δ=1.

However, if ValueOfImplementation_retention_ <0, then the value of additional research is estimated as:

VOI_retention_= $|\frac{\sum_{k=1}^{k} \left( Effectivenessk \right|Effectivenessk>0)}{k}$| * P (Strategy Effective) *δ

if ValueOfImplementation_retention_ $\leq$0

, where δ∈(0,1) and we assume that for all retention strategies, δ=1.
