# Supplementary material for: Update to Trial Forge Guidance 2: addition of the Value of Information criterion
Source: Trials. 2026 Jan 24;27:141. doi: 10.1186/s13063-026-09443-7 (PMC12910824; doi:10.1186/s13063-026-09443-7)
Supplement: Supplementary file 2 — Supplementary Material 2. [file 13063_2026_9443_MOESM2_ESM.docx]

# **Supplementary Material 2: Value of Information feedback**

Feedback was received from one trial methodologist, three trial managers and one funder of trials.

**User feedback on the draft decision aid tool**

Overall, the decision aid tool was positively received. Participants described it as an excellent idea and a potentially valuable addition to existing trial methodology and SWAT guidance. Several respondents suggested that, once finalised, the tool could be referenced in funder guidance documents to support applicants planning SWATs.

Ease of use was rated as moderate, with a mean score of **3 out of 5** (five indicating very easy). Participants highlighted the need for clearer, step-by-step instructions and a worked example to facilitate use. It was noted that researchers and clinicians with experience in trials and methodological research may find the tool easier to use. Additional feedback concerned the underlying database used for the drop-down menu options, including clarification of how this would be maintained and updated over time.

The feedback form is shown on Page 2.

# Trial forge guidance tool feedback form

**After reviewing the Trial Forge Guidance tool, we would be grateful if you could complete the questions below.**

**Your job role (Please tick one)**

I represent a funder of trials I am a trialist or trial methodologist

**How would you describe your sex? (Please tick one)**

Male Female Other I do not wish to state

1. **What is your first impression of the tool?**
2. **How easy or difficult is it to use the VoI tool? (Please circle one)**

| Very difficult | 1 | 2 | 3 | 4 | 5 | Very easy |
| --- | --- | --- | --- | --- | --- | --- |

1. **Is there anything you would change about how the tool works?**
