# Supplementary material for: Update to Trial Forge Guidance 2: addition of the Value of Information criterion
Source: Trials. 2026 Jan 24;27:141. doi: 10.1186/s13063-026-09443-7 (PMC12910824; doi:10.1186/s13063-026-09443-7)

**Supplementary Material 3: Meta-analysis forest plots**


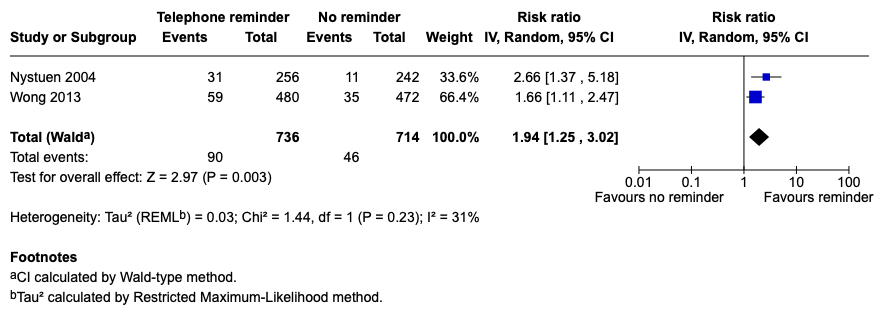
Example 1: Telephone reminder versus no telephone reminder (recruitment strategy)


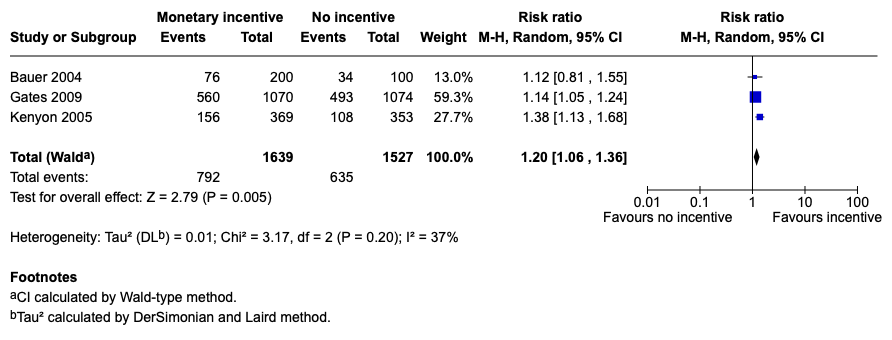
Example 2: Monetary incentive versus no monetary incentive (retention strategy)

Example 3: Optimised information versus standard information (retention strategy)


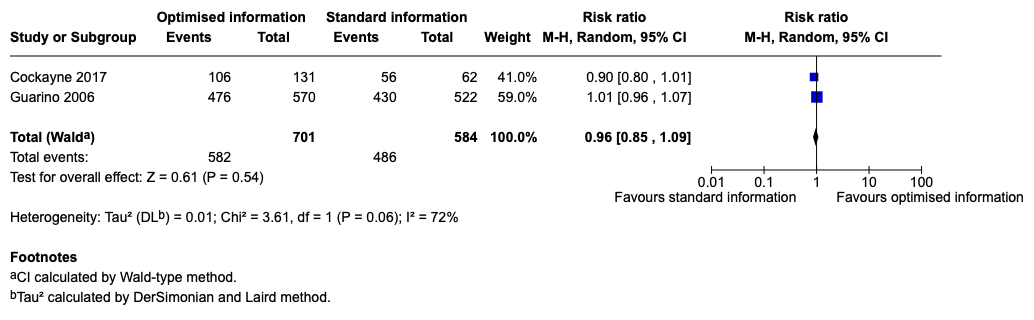

Supplement: Supplementary file 3 — Supplementary Material 3. [file 13063_2026_9443_MOESM3_ESM.docx]
